# Headache frequency and neck pain are associated with trapezius muscle T2 in tension-type headache among young adults

**DOI:** 10.1186/s10194-023-01626-w

**Published:** 2023-07-12

**Authors:** Nico Sollmann, Paul Schandelmaier, Dominik Weidlich, Jonathan Stelter, Gabby B. Joseph, Corinna Börner, Severin Schramm, Meinrad Beer, Claus Zimmer, Mirjam N. Landgraf, Florian Heinen, Dimitrios C. Karampinos, Thomas Baum, Michaela V. Bonfert

**Affiliations:** 1 Department of Diagnostic and Interventional Neuroradiology, School of Medicine, Klinikum rechts der Isar, Technical University of Munich, Munich, Germany; 2 TUM-Neuroimaging Center, Klinikum rechts der Isar, Technical University of Munich, Munich, Germany; 3 Department of Diagnostic and Interventional Radiology, University Hospital Ulm, Ulm, Germany; 4 Department of Radiology and Biomedical Imaging, University of California San Francisco, San Francisco, CA USA; 5 Department of Pediatrics – Dr. von Hauner Children’s Hospital, Division of Pediatric Neurology and Developmental Medicine, LMU Hospital, Ludwig-Maximilians-Universität München, Munich, Germany; 6 LMU Center for Children with Medical Complexity – iSPZ Hauner, Ludwig-Maximilians-Universität München, Munich, Germany; 7 Department of Diagnostic and Interventional Radiology, Klinikum rechts der Isar, School of Medicine, Technical University of Munich, Munich, Germany

**Keywords:** Magnetic resonance imaging, Migraine, Tension-type headache, Trapezius muscle, T2 mapping, Trigemino-cervical complex

## Abstract

**Background:**

Tension-type headache (TTH) is the most prevalent primary headache disorder. Neck pain is commonly associated with primary headaches and the trigemino-cervical complex (TCC) refers to the convergence of trigeminal and cervical afferents onto neurons of the brainstem, thus conceptualizes the emergence of headache in relation to neck pain. However, no objective biomarkers exist for the myofascial involvement in primary headaches. This study aimed to investigate the involvement of the trapezius muscles in primary headache disorders by quantitative magnetic resonance imaging (MRI), and to explore associations between muscle T2 values and headache frequency and neck pain.

**Methods:**

This cohort study prospectively enrolled fifty participants (41 females, age range 20–31 years): 16 subjects with TTH only (TTH-), 12 with mixed-type TTH plus migraine (TTH+), and 22 healthy controls (HC). The participants completed fat-suppressed T2‐prepared three-dimensional turbo spin-echo MRI, a headache diary (over 30 days prior to MRI), manual palpation (two weeks before MRI), and evaluation of neck pain (on the day of MRI). The bilateral trapezius muscles were manually segmented, followed by muscle T2 extraction. Associations between muscle T2 and the presence of neck pain as well as the number of days with headache (considering the 30 days prior to imaging using the headache calendar) were analyzed using regression models (adjusting for age, sex, and body mass index).

**Results:**

The TTH+ group demonstrated the highest muscle T2 values (right side: 31.4 ± 1.2 ms, left side: 31.4 ± 0.8 ms) as compared to the TTH- group or HC group (*p* = 0.011). Muscle T2 was significantly associated with the number of headache days (β-coefficient: 2.04, *p* = 0.04) and the presence of neck pain (odds ratio: 2.26, *p* = 0.04). With muscle T2 as the predictor, the area under the curve for differentiating between HC and the TTH+ group was 0.82.

**Conclusions:**

Increased T2 of trapezius muscles may represent an objective imaging biomarker for myofascial involvement in primary headache disorders, which could help to improve patient phenotyping and therapy evaluation. Pathophysiologically, the increased muscle T2 values could be interpreted as a surrogate of neurogenic inflammation and peripheral sensitization within myofascial tissues.

## Introduction

Primary headaches belong to the most prevalent neurological disorders worldwide and are associated with high morbidity and restrictions in quality of life [1, 2]. While pain localized within the head is considered the cardinal symptom of tension-type headache (TTH) as well as migraine, the spectrum of symptoms frequently includes neck pain [2–5]. Specifically, neck pain was identified to be at least as common as nausea as a major accompaniment of migraine attacks, has shown a very high prevalence in people with TTH, and is associated with headache chronicity [2, 5, 6].

The etiology of TTH as well as migraine is multifactorial, and recent pathophysiological concepts converge central and peripheral mechanisms of pain perception, processing, perpetuation, and sensitization [2, 7, 8]. For the peripheral component, complaints such as neck pain and findings such as tension, generalized or as focal hypersensitivity, taut bands, and referred sensation/pain at the neck play a major role [2, 7]. Nociception from myofascial structures is mediated by thin myelinated (Aδ) and unmyelinated (C) fibers that are activated by stimuli such as muscle contraction or strain, ischemia, or inflammation [2, 7–11]. Stress can trigger headache by increasing neck muscle tension and by exaggerating the activity of motor units inducing ischemia-like states, which can consecutively entail increased responsiveness of the terminal nerve endings of the Aδ and C fibers (peripheral sensitization) [2, 7–11]. Peripheral sensitization can be enhanced by vasoactive and neurogenic mediators, such as calcitonin gene-related peptide (CGRP) that causes vasodilation and mast cell degradation, leading to plasma exudation in the myofascial tissue [2, 5, 7–13]. The nociceptive sensation at the neck is mostly conveyed via the Aδ and C fibers running within the C1 to C3 afferents to the trigemino-cervical nucleus and is further processed with trigeminal afferent inputs, representing the trigemino-cervical complex (TCC) [2, 7, 8, 14]. Here, the information is transmitted onto second-order neurons and further transferred to the trigemino-thalamic tract and linked to the central pain processing regions [2, 7, 9–11]. The extent of nociceptive information forwarded depends on the level of sensitization of the second-order and higher-order neurons (central sensitization) and on the level of top-down inhibition from cortical regions, the hypothalamus, periaqueductal gray, and the brainstem [2, 7, 8, 13]. The nociceptive inflow to the trigemino-cervical nucleus may further trigger neurogenic inflammation by retrograde excretion of CGRP, and, thus, the process of peripheral sensitization is continuously exaggerated and perpetuated.

According to the International Classification of Headache Disorders (ICHD) Edition-3, neck pain is not part of the current diagnostic criteria for primary headaches [15]. Both TTH and migraine are defined solely by clinical criteria, translating into recent interest in establishing objective biomarkers for patient phenotyping and precision medicine [2, 7, 8, 15]. However, to date, no objective biomarker has been introduced particularly for myofascial involvement in primary headache disorders, and this lack may favor suboptimal treatment, erroneous diagnosis, and impedes predicting the disease course [2, 7, 8]. Thus, the reference standard for investigating muscular involvement in primary headaches including the detection of myofascial trigger points (mTrPs) is still given by manual palpation of muscles [16]. Yet, this approach can obviously be questioned with respect to reproducibility and reliability. However, quantitative magnetic resonance imaging (MRI) could enable identifying changes of skeletal musculature towards definition of objective biomarkers [17–19]. Specifically, MRI with T2 mapping could serve as a method to detect and quantify changes in muscles related to neurogenic inflammation in primary headaches, hence it could provide measures representative of peripheral sensitization at myofascial structures. In this context, one previous study has demonstrated that T2 values of the trapezius muscles were significantly higher in subjects with migraine as compared to healthy controls (HC) [18]. Yet, it remains to be elucidated whether elevated muscle T2 could also be detected in TTH, and whether it is associated with clinically determined neck pain and headache characteristics.

The trapezius muscle as one of the largest skeletal muscles with a superficial location has recently seen particular attention regarding the myofascial involvement in primary headache disorders. The upper trapezius muscle is easily accessible to manual examination, and investigations were successful in provoking headache attacks by manual palpation delivered to mTrPs of the trapezius muscles [20]. It is innervated by anterior rami of cervical spinal nerves, thus connects to the concept of the TCC [2, 7, 8, 14]. From a treatment perspective, previous work has chosen the trapezius muscle as the target for repetitive neuromuscular magnetic stimulation (rNMS) to treat headache and local muscular hyperalgesia, with the TCC functioning as a potential crosslink between peripheral stimulation and central alleviation of headache [21–25]. Hence, given its innervation profile and role within the TCC, the trapezius muscle may be a structure of particular interest to investigate myofascial involvement and peripheral sensitization in primary headaches.

Against this background, the aim of this study was to investigate the trapezius muscles in patients with primary headaches with quantitative MRI using T2 mapping. We hypothesized that patients with TTH show increased muscle T2 values compared to HC as a surrogate of the myofascial involvement triggered by neurogenic inflammation, and that muscle T2 values are associated with neck pain and headache frequency.

## Methods

### Ethics and consent

This prospective monocentric study was approved by the local ethics committee and conducted in accordance with the Declaration of Helsinki (registration numbers: 154–12 & 5679/13 & 193/19 S, Ethikkommission der Technischen Universität München). Written informed consent was a prerequisite for study participation.

### Study design and eligibility criteria

Inclusion criteria were 1) age of at least 18 years; 2) a diagnosis of TTH only (TTH- group), a diagnosis of mixed-type headache (TTH and migraine, TTH+ group), or absence of any history of headache disorders in HC. Classification of headache disorders was done according to the diagnostic criteria of the ICHD Edition-3 [15]. The classifications were confirmed by considering the headache diary of the German Migraine and Headache Society (DMKG) ([26], https://www.dmkg.de/files/Kopfschmerzkalender_PDF/Kopfschmerzkalender_ENGLISCH_18.3.2021.pdf). Exclusion criteria were 1) any history of muscular or neurological disorders (except for the respective headache diagnosis); 2) a diagnosis of migraine only or a diagnosis of any other primary headaches (e.g., cluster headache); 3) any history of previous injury, surgery, or implants at the neck region; 4) participation in competitive sports, extensive physical activity, or weightlifting/body building; 5) intake of muscle relaxers; 6) any interventions for neck pain such as massage or physiotherapy (at least during the two weeks prior to MRI); 7) a body mass index (BMI) indicating underweight or obesity (BMI < 18.5 or BMI > 30.0 kg/m^2^); 8) contraindications for MRI acquisition; 9) pregnancy.

The study protocol included manual palpation of the neck muscles, assessment of the presence of neck pain and headache frequency, and acquisition of MRI of the neck region. A two-weeks interval was considered between manual palpations and MRI acquisitions. 

### Manual palpation

The assessment of the upper trapezius muscles was performed by manual palpation by a certified physiotherapist [27, 28]. The examiner let the trapezius muscle slide through between thumb and index finger (pincer grip) under medium pressure. If present, taut bands and mTrPs and their locations were documented. The entire course of the upper trapezius muscles was assessed bilaterally, and the following criteria had to be fulfilled for a latent mTrP: 1) palpable taut band with a local hypersensitive spot; 2) local hypersensitive spot with occurrence of a referred sensation during palpation; or 3) palpable taut band with a local hypersensitive spot and occurrence of a referred sensation during palpation [27–29]. For an active mTrP, the referred sensation of a hypersensitive spot during palpation was required to reproduce the individual typical headache symptoms [27–30]. The total number of mTrPs, if any, was documented for the left and right trapezius muscles, respectively.

### Assessment of headache and neck pain

On a daily basis for an interval of 30 days before the day of the MRI acquisition, the headache diary of the DMKG had to be filled in to record the average number of days with headache per month ([26], https://www.dmkg.de/files/Kopfschmerzkalender_PDF/Kopfschmerzkalender_ENGLISCH_18.3.2021.pdf). We also documented whether a study participant subjectively suffered from neck pain on the day of MRI acquisition. In this context, neck pain was defined as pain in the cervical spine region (with or without pain referred to the arms) that lasted for at least 24 hours [31].

### Magnetic resonance imaging

#### Image acquisition

Scanning of the neck and shoulder region was performed with a 3-Tesla MRI scanner (Ingenia Elition, Philips Healthcare, Best, The Netherlands) in supine position using a 16-channel anterior coil, 12-channel built-in-table posterior coil, and 16-channel head coil. All patients with TTH or a concomitant diagnosis of TTH and migraine were investigated in their inter-ictal phases. The detailed pulse sequence parameters of the T2‐prepared three-dimensional (3D) turbo spin-echo (TSE) sequence for T2 mapping are shown in Table 1. For anatomical co-registration, a T2-weighted DIXON TSE sequence covering the same anatomical location was acquired.

**Table 1:** Imaging protocol

**Sequence description**	T2‐prepared three-dimensional (3D) turbo spin-echo (TSE)
Fat suppression	Spectral inversion recovery
Repetition time	1500 ms
Echo time	16 ms
Field of view (FOV)	480 × 200 × 84 mm^3^
Acquisition voxel	1.75 × 1.75 × 2.0 mm^3^
Reconstruction voxel	1.5 × 1.5 × 2.0 mm^3^
Echo train length	55
Echo spacing	2.3 ms
Compressed sensitivity encoding	SENSE, reduction factor *R* = 5.5
Partial Fourier	None
T2 preparation	15 – 30 – 45 ms
Acquisition time	7 min 53 s

This table shows the pulse sequence parameters for 3-Tesla magnetic resonance imaging (MRI) of the neck and shoulder region. The same MRI machine and sequence was used in subjects with tension-type headache (TTH-), TTH plus migraine episodes (TTH+), and healthy controls (HC). The images at the different T2 preparations were reconstructed online using the reconstruction software of the scanner combining SENSE and compressed sensing. An additional saturation preparation scan was acquired to limit errors due to B0 inhomogeneities [17]. The flip angle train was chosen according to the vendor's routines, establishing a constant signal over the entire shot for the relaxation properties of skeletal musculature [17–19]

#### Image segmentation and T2 extraction

Processing of raw image data was performed using in-house developed scripts for MATLAB (version R2021a; MathWorks Inc., Natick, MA, USA), and visual quality assessment followed by segmentation was performed using image viewer software (MITK, version 2022.04; www.mitk.org). A voxel-by-voxel fitting with additional accounting for B0 field inhomogeneities was applied [17–19].

**Fig. 1:**
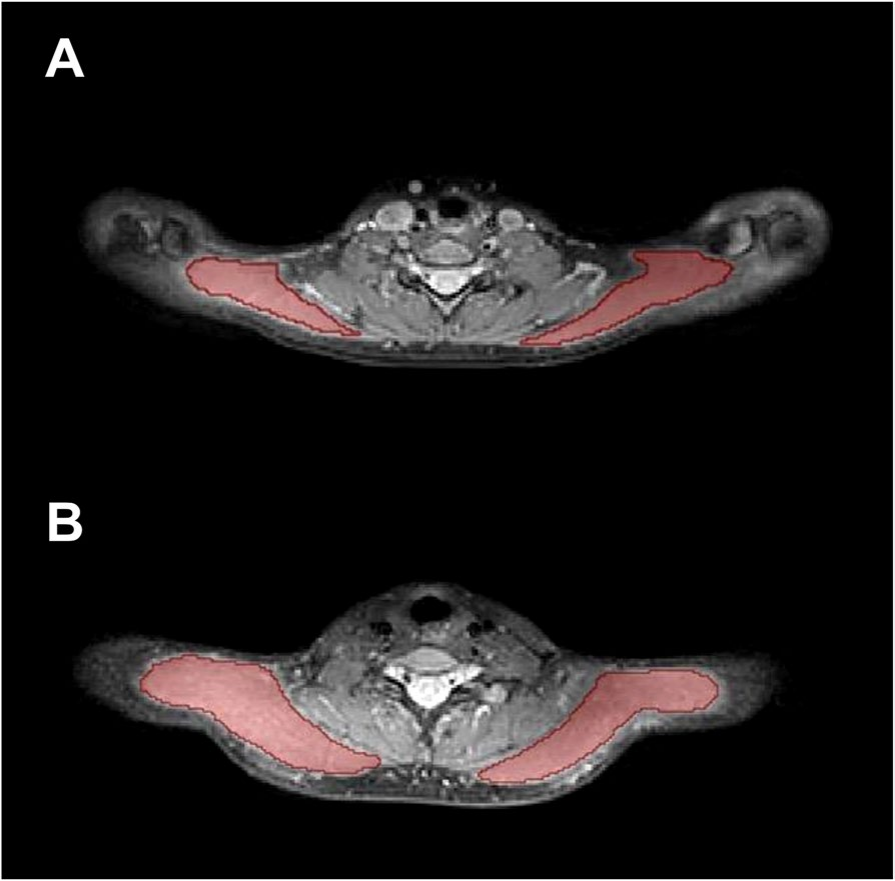
Exemplary cases for trapezius muscle segmentations. Segmentation masks of the bilateral trapezius muscles (red areas) in a 25-year-old female (body mass index [BMI] = 20.1 kg/m^2^; **A**) and in a 24-year-old male (BMI = 19.9 kg/m^2^; **B**)

In the axial slices of the images with the shortest T2 preparation duration, the complete trapezius muscles were manually segmented bilaterally (Fig. 1). Polygonal regions of interest (ROIs) were drawn to enclose the entire right and left upper trapezius muscles [19]. To prevent inadvertent inclusion of muscle fascia or intermuscular fat, a margin of about 5 mm was kept from the outer contour of the trapezius muscles. In vertical direction, the segmentation ended in the transition area when muscle tendons appeared in place of muscle tissue. Using the MATLAB scripts, the mean T2 values of the left and right trapezius muscles were extracted, while values > 100 ms were excluded (given that such high T2 values were most likely stemming from areas of high fluid components, i.e. vasculature) [19, 32]. All image segmentations were done by one reader, who was blinded to the results of physical examination as well as the group assignments (HC, TTH-, and TTH+ groups).

The reproducibility of manual segmentations with T2 extraction for the trapezius muscles has been shown to be high, with a root-mean-square coefficient of variation (RMSCV) of 0.12 ± 0.07% (range of RMSCV: 0.01–0.23%), as well as high inter-reader reliability with an RMSCV of 1.43 ± 0.64% (range of RMSCV: 0.90–2.50%) [19].

### Statistical analysis

Statistical analysis was performed using STATA (version 16; StataCorp LP, College Station, TX, USA), Excel (2019 MSO, version 2410; Microsoft Corp., Redmond, WA, USA), and Prism (version 6; GraphPad Prism, San Diego, CA, USA). Descriptive statistics were calculated for study participant characteristics (i.e., age, sex, and BMI) as well as headache-related characteristics (number of mTrPs in trapezius muscles, medication intake, number of days with headache, and presence of neck pain at the day of scanning). Differences in continuous parameters (i.e., age, BMI, number of mTrPs in trapezius muscles, medication intake, and number of days with headache) between groups (HC, TTH-, and TTH+ groups) were assessed using t-tests, and differences in categorical parameters between groups (i.e., sex and presence of neck pain) were assessed using Chi-squared tests. Furthermore, mean ± standard deviation (SD) and ranges were calculated for muscle T2 values, using the right-sided, left-sided, and average values between both sides.

Associations between muscle T2 values (mean of left and right side) and the outcome of number of days with headache that did not vary by body side were analyzed using linear regression models. For the binary outcome of neck pain (which also did not vary by body side), logistic regression was performed with average muscle T2 as predictor. In outcomes that varied by body side (average number of mTrPs according to manual palpation), mixed effects models were utilized to assess the associations with muscle T2. All models were adjusted for age, sex, and BMI. Adjusted β-coefficients, odds ratio (OR), *p*-values, and/or 95%-confidence intervals (95%-CIs) are reported for these models.

Furthermore, the association between average muscle T2 and group (HC, TTH-, and TTH+ groups) was evaluated using logistic regression. Specifically, three logistic regression models were performed with the following outcomes: HC vs. TTH-, HC vs. TTH+ , and TTH- vs. TTH+ groups. In addition, associations between muscle T2 and side in each group were analyzed. For differentiation between groups, the area under the curve (AUC) was calculated. For all statistical testing, a *p*-value < 0.05 was considered statistically significant.

## Results

### Participant characteristics

Overall, 50 participants were included in this study: 16 participants with TTH only (TTH-), 12 participants with a mixed-type headache of TTH and migraine episodes (TTH+), and 22 HC. The TTH- as well as the TTH+ groups did not significantly differ from the HC group with respect to age, sex, or BMI (Table 2). Participants of the TTH+ group had on average 2.4 ± 1.8 migraine episodes during the month prior to the day of the MRI acquisitions.

**Table 2:** Participant characteristics

	**HC (*n* = 22)**	**vs. TTH- (*n* = 16)**	***p*-value**	**vs. TTH+ (*n* = 12)**	***p*-value**
**Sex** no. females	17 (77%)	12 (75%)	0.870	12 (100%)	0.073
**Age (years)** mean ± SD, range	23.0 ± 2.2 19.7 – 28.1	24.8 ± 3.4 20.9 – 31.0	0.077	23.6 ± 3.4 20.6 – 29.9	0.583
**BMI (kg/m**^**2**^**)** mean ± SD, range	22.0 ± 2.3 19.1 – 28.7	22.1 ± 2.4 18.8 – 28.1	0.843	21.7 ± 1.7 18.9 – 24.7	0.689
**No. of mTrPs** mean ± SD, range	4.0 ± 4.0 0 – 10	3.8 ± 2.8 0 – 10	0.898	3.8 ± 2.0 1 – 8	0.906
**Medication intake** **(days/month)** mean ± SD, range	1.0 ± 1.5 0 – 5	3.8 ± 4.5 0 – 18	***0.027***	4.4 ± 2.8 0 –11	***0.002***
**Headache (days/month)** mean ± SD, range	2.0 ± 1.5 0 - 5 (*n*=21)	10.1 ± 7.6 3 – 30	** < *0.001***	10.3 ± 6.8 4 – 25	***0.001***
**Neck pain** no. subjects	0 (0%)	10 (63%)	** < *0.001***	10 (83%)	** < *0.001***

This table shows the characteristics of the study sample. Statistical analysis aimed to assess for statistically significant differences regarding sex, age, body mass index (BMI), number of myofascial trigger points (mTrPs), medication intake (analgesic drugs, days/month), headache (days/month), and neck pain (number of subjects with neck pain) in subjects with tension-type headache only (TTH-), TTH plus migraine episodes (TTH+), and healthy controls (HC). Values are given as absolute numbers or percentages, mean values ± standard deviation (SD), and/or ranges

### T2 values of the trapezius muscles

The HC group demonstrated the lowest T2 values (right side: 30.0 ± 1.1 ms, left side: 30.2 ± 1.1 ms), followed by the TTH- group (right side: 30.8 ± 1.1 ms, left side: 31.1 ± 1.2 ms) and the TTH+ group (right side: 31.4 ± 1.2 ms, left side: 31.4 ± 0.8 ms). Differences between body sides were not statistically significant in all three groups (*p* > 0.05). However, statistically significant associations for the average T2 value (mean of left- and right-sided trapezius muscles) and group assignments were observed (HC vs. TTH-: *p* = 0.048; HC vs. TTH+ : *p* = 0.011; TTH- vs. TTH+ : *p* = 0.239; delta method: HC / TTH- / TTH+: *p* < 0.001).

### Associations between muscle T2 values with clinical parameters

The average T2 value (mean of left- and right-sided trapezius muscles) was significantly associated with the number of days with headache (β-coefficient: 2.04, 95%-CI: 0.05–4.03, *p* = 0.04). Thus, for every day increase in the number of days with headache, the T2 value would increase by 2.04 ms. Furthermore, the average T2 value was significantly associated with the presence of neck pain (OR: 2.26, 95%-CI: 1.04–4.90, *p* = 0.04). Yet, average T2 values were not statistically significantly associated with the number of mTrPs as defined by manual palpation (β-coefficient: 0.07, 95%-CI: -0.22–0.37, *p* = 0.62).

### Group differentiation based on T2 values

With average T2 as the predictor, the AUC for differentiating between the HC and TTH- groups was 0.68 (OR: 2.03, 95%-CI: 1.01–4.11), and it was 0.82 for the differentiation between the HC and TTH+ groups (OR: 3.80, 95%-CI: 1.36–10.61). Furthermore, the AUC for differentiating between the TTH- and TTH+ groups was 0.69 (OR: 1.61, 95%-CI: 0.73–3.53).

## Discussion

This study used T2 mapping to investigate the trapezius muscles in patients with TTH in comparison to HC. The main findings were as follows: 1) significant associations for average muscle T2 values and group assignments (HC, TTH-, and TTH+) were observed, with patients affected by mixed-type TTH plus migraine episodes demonstrating the highest muscle T2; 2) average T2 values were significantly associated with the number of days with headache and the presence of neck pain; 3) muscle T2 values could allow differentiating between HC and patients suffering from TTH plus migraine (with an AUC of 0.82).

The TCC provides a concept for interconnecting peripheral and central mechanisms in headache pathophysiology, with increased nociceptive input from the neck musculature (e.g., the upper trapezius muscle as the largest representative) being conveyed via the trigemino-cervical nucleus to higher-order pain processing regions [2, 7, 9–11, 14]. Regarding the role of myofascial structures, there is evidence for activation and sensitization of nociceptors by local mechanisms induced by ischemic-like states caused by exaggerated activity of motor units triggering the excretion of allogenic mediators, and by neurogenic inflammatory mediators such as CGRP that is released into the tissue [2, 7, 9–12, 33]. Additionally, CGRP can affect muscle function as it can modulate the actions of acetylcholine at the neuromuscular junction [12, 34]. In this regard, proinflammatory substances have been observed with elevated concentrations specifically in the trapezius muscles in subjects with active mTrPs, which are highly prevalent in primary headaches [35–39]. Furthermore, patients with TTH showed increased pain sensitivity after intramuscular infusion of inflammatory substances relating to sensitization, which is most likely caused by released endogenous inflammatory mediators [2, 40]. Edema typically follows inflammatory processes as vasoactive mediators promote plasma exudation, which can be captured in general by MRI of muscular tissue [41, 42]. However, inflammation with edematous changes may not be directly visible, given that changes might be subtle in primary headache disorders. Yet, the herein used T2 mapping approach may enable detecting and objectively quantifying muscle T2 as a surrogate of subtle edematous changes in response to inflammation and/or ischemia. This may be represented by the increased muscle T2 values of patients with TTH and patients with a concomitant diagnosis of TTH and migraine when compared to HC. Specifically, the highest values for muscle T2 were observed in patients with a concomitant diagnosis of TTH and migraine. In this context, previous work using self-reported data or provoked muscular pain indicated that neck pain is more prevalent in patients with TTH plus migraine, followed by patients with TTH only and HC [3, 4]. Furthermore, it has been shown that neck pain was positively associated with coexisting TTH in patients with migraine [43]. Hence, T2 mapping may support those findings with an objective and quantitative parameter of the trapezius muscles.

Furthermore, in this study, muscle T2 values were significantly associated with the number of days with headache and the presence of neck pain, thus providing evidence for a link between pain perceived peripherally and centrally and findings from T2 mapping. It has previously been demonstrated that neck pain disability derived from assessments with the Neck Disability Index (NDI) was associated with the frequency of migraine attacks, adding to the overall disability in episodic and chronic migraine [6]. Further, the strength of associations between self-reported neck pain and migraine increased with the frequency of migraine days, with patients suffering from a high attack frequency (at least 15 days per month) showing the most pronounced associations [44]. Likewise, headache-related impairment among patients with migraine was significantly predictive of neck pain measured with the NDI, as were neck pain intensity and frequency [45]. However, in lack of a biomarker for associations between headache frequency and neck pain, those findings could not be supported by measurable correlates beyond self-reporting or manual investigations [6, 44, 45]. In this regard, T2 mapping could be applicable to support observations of the interconnection between symptoms on the central and muscular level by non-invasive and objectifiable measures. In this study, T2 mapping was realized by a high-resolution T2-prepared 3D TSE sequence that can provide accurate and fast T2 quantification with sufficient robustness to B1 and B0 errors, which seems particularly important for a challenging area such as the neck region that can be characterized by large B0 variations [17–19]. Compared to more commonly applied T2 mapping approaches using a multi-echo spin-echo (MESE) sequence, this approach may overcome issues related to the dependence of the T2 quantification on B1 and B0 errors [19, 46, 47].

Recent efforts have been spent on including impaired neck function or pain as a criterion for stratifying between patients and headache subtypes [43, 48, 49]. Although highly prevalent, neck pain is not part of the current diagnostic criteria of the ICHD [15]. Yet, its high prevalence in patients with primary headaches makes it a relevant phenomenon that could contribute to improved patient phenotyping and individualizing treatment regimens and monitoring. Differentiation between HC and patients affected by TTH plus migraine based on muscle T2 acquired during the inter-ictal state was possible with an AUC of 0.82, which may have implications for diagnostics and therapy. In this regard, one study indicated that migraine patients with ictal neck pain have increased neck muscle tenderness interictally, interpreted as sign of peripheral sensitization even in-between acute migraine attacks [43]. This may in part explain our findings of elevated muscle T2 in the inter-ictal phases and the possibility to perform patient stratification based on T2 mapping, but could also provide a further rationale for preventive treatment approaches targeting the neck musculature. It has been demonstrated that repeated sessions of rNMS delivered to the upper trapezius muscles during the inter-ictal phase of patients suffering from migraine could reduce the frequency and intensity of migraine attacks, alongside with relief of hyperalgesia at the neck region [21–25]. As such, rNMS could non-invasively address neck pain and reduce the underpinned peripheral sensitization of the nociceptive Aδ and C fibers within the targeted myofascial structures. By these effects, trigeminal and central pain mechanisms involved in primary headache disorders could be modulated via the TCC [50]. Another treatment applied at the myofascial level is injection of Onabotulinumtoxin A, which blocks releases of CGRP and other neuropeptides in the myofascial tissue, thus limiting the level of peripheral sensitization and therefore reducing the afferent flow delivered by the first trigeminal branch into the TCC [51, 52]. In this regard, T2 mapping may help to monitor effects of therapy as it could be highly sensitive to changes over time due to therapy applied at the muscular level.

Besides a potential role for monitoring of therapy effects, T2 mapping may be used to evaluate the degree of convergent validity of findings from manual palpation and derived mTrPs. One previous study has investigated signal alterations in T2 maps attributed to mTrPs in a small cohort of subjects with migraine, revealing increased T2 values at sites of manually determined mTrPs in the trapezius muscles [18]. Hence, T2 mapping of the trapezius muscles could potentially challenge the current reference standard method of physical examination of mTrPs, and could allow for more targeted and objective definitions of mTrPs [18]. While average T2 values of the segmented trapezius muscles were not significantly associated with the mere number of mTrPs as defined by manual palpation according to the findings of the present study, T2 mapping may be used as a distinct targeting tool to identify mTrPs by means of focally increased T2 values, which could help to objectively localize mTrPs. Furthermore, potential associations between findings from ultrasound and T2 mapping need to be elucidated. Given that T2 mapping derived from MRI for the assessment of muscular involvement in primary headaches might not be accessible for daily clinical practice due to costs and limited availability, ultrasound as a point-of-care alternative for this purpose may have a role if findings could be correlated to the more standardized measures from quantitative MRI.

A major limitation of this study is the small sample size. Second, although all subjects with TTH or TTH plus migraine were investigated in their inter-ictal phases, the distinct interval to the previous headache event has not been considered. Third, the AUC for differentiating between HC, TTH-, or TTH+ is not yet sufficient for wider use, and future studies need to refine AUCs in larger trials. It should be emphasized that the etiology of TTH as well as migraine is multifactorial and using only one measure (e.g., T2 values) for patient stratification may not reach very high AUC values in any case. Thus, integrative models that use several and ideally objectifiable parameters and biomarkers for improved stratification are highly warranted, and muscle T2 could be one of those in the future. Fourth, we did not investigate potential associations between T2 values and the intensity of headache and neck pain in this study, although such associations may also contribute to further exploration of muscle T2 as a potential biomarker of myofascial involvement and peripheral sensitization in primary headaches.

## Conclusions

Using T2 mapping with a 3D T2-prepared TSE sequence, this study found significant associations of muscle T2 and group assignments for primary headache disorders (TTH, mixed-type of TTH plus migraine) and HC. Furthermore, muscle T2 was significantly positively associated with headache frequency and the presence of neck pain. Pathophysiologically, it could be interpreted as a surrogate of neurogenic inflammation and peripheral sensitization within myofascial structures.

## Data Availability

The datasets used and/or analysed during the current study are available from the corresponding author on reasonable request.

## Abbreviations

3D: Three-dimensional
AUC: Area under the curve
BMI: Body mass index
CGRP: Calcitonin gene-related peptide
CI: Confidence interval
DMKG: German Migraine and Headache Society
FOV: Field of view
HC: Healthy controls
ICHD: International Classification of Headache Disorders
MESE: Multi-echo spin-echo
MRI: Magnetic resonance imaging
NDI: Neck Disability Index
mTrP: Myofascial trigger point
OR: Odds ratio
RMSCV: Root-mean-square coefficient of variation
rNMS: Repetitive neuromuscular magnetic stimulation
ROI: Region of interest
SD: Standard deviation
SENSE: Sensitivity encoding
TCC: Trigemino-cervical complex
TSE: Turbo spin-echo
TTH: Tension-type headache
