# Correction: Headache frequency and neck pain are associated with trapezius muscle T2 in tension‑type headache among young adults

**DOI:** 10.1186/s10194-025-01964-x

**Published:** 2025-02-20

**Authors:** Nico Sollmann, Paul Schandelmaier, Dominik Weidlich, Jonathan Stelter, Gabby B. Joseph, Corinna Börner, Severin Schramm, Meinrad Beer, Claus Zimmer, Mirjam N. Landgraf, Florian Heinen, Dimitrios C. Karampinos, Thomas Baum, Michaela V. Bonfert

**Affiliations:** 1 Department of Diagnostic and Interventional Neuroradiology, School of Medicine, Klinikum rechts der Isar, Technical University of Munich, Munich, Germany; 2 TUM-Neuroimaging Center, Klinikum rechts der Isar, Technical University of Munich, Munich, Germany; 3 Department of Diagnostic and Interventional Radiology, University Hospital Ulm, Ulm, Germany; 4 Department of Radiology and Biomedical Imaging, University of California San Francisco, San Francisco, CA USA; 5 Department of Pediatrics– Dr. von Hauner Children’s Hospital, Division of Pediatric Neurology and Developmental Medicine, LMU Hospital, Ludwig-Maximilians-Universität München, Munich, Germany; 6 LMU Center for Children with Medical Complexity - iSPZ Hauner, Ludwig-Maximilians-Universität München, Munich, Germany; 7 Department of Diagnostic and Interventional Radiology, Klinikum rechts der Isar, School of Medicine, Technical University of Munich, Munich, Germany

**Correction: J Headache Pain 24**,** 84 (2023).**

10.1186/s10194-023-01626-w

Following publication of this article, the authors identified some errors in the text and Table 2 that resulted in an incorrect representation of the data and may eventually lead to misinterpretations of parts of the findings of this article.

The respective correction of those issues does not change the main direction or conclusions of the article.

The corrected text, along with the affected rows and columns of Table 2 are highlighted below with bold font and the original article has been updated.

The authors would like to apologize for any inconvenience caused to the readers.

**Table:** 

Section	Page	Incorrect	Correct
Abstract (Results)	1	The TTH + group demonstrated the highest muscle T2 values (right side: 31.4 ± 1.2 ms, left side: 31.4 ± 0.8 ms) as compared to the TTH- group or HC group **(***p* < 0.001).	The TTH + group demonstrated the highest muscle T2 values (right side: 31.4 ± 1.2 ms, left side: 31.4 ± 0.8 ms) as compared to the TTH- group or HC group **(***p*** = 0.011).**
Statistical analysis	4	Statistical analysis was performed using STATA (version 16; StataCorp LP, College Station, TX, USA) and Prism (version 6; GraphPad Prism, San Diego, CA, USA).	Statistical analysis was performed using STATA (version 16; StataCorp LP, College Station, TX, USA), **Excel (2019 MSO**,** version 2410; Microsoft Corp.**,** Redmond**,** WA**,** USA)**, and Prism (version 6; GraphPad Prism, San Diego, CA, USA).
T2 values of the trapezius muscles	5	The HC group demonstrated the lowest T2 values (right side: 30.0 ± 1.1 ms, left side: 30.2 ± 1.1 ms), followed by the TTH- group (right side: 30.8 ± 1.1 ms, left side: 30.9 ± 1.1 ms) and the TTH + group (right side: 31.4 ± 1.2 ms, left side: 31.4 ± 0.8 ms).	The HC group demonstrated the lowest T2 values (right side: 30.0 ± 1.1 ms, left side: 30.2 ± 1.1 ms), followed by the TTH- group (right side: 30.8 ± 1.1 ms, left side: **31.1 ± 1.2** ms) and the TTH + group (right side: 31.4 ± 1.2 ms, left side: 31.4 ± 0.8 ms).
T2 values of the trapezius muscles	5	However, statistically significant associations for the average T2 value (mean of left- and right-sided trapezius muscles) and group assignments were observed (HC vs. TTH-: *p* < 0.001; HC vs. TTH+: *p* < 0.001; TTH- vs. TTH+: *p* < 0.001).	However, statistically significant associations for the average T2 value (mean of left- and right-sided trapezius muscles) and group assignments were observed **(HC vs. TTH-**: ***p*** **= 0.048; HC vs. TTH+**: ***p*** **= 0.011; TTH- vs. TTH+**: ***p*** **= 0.239; delta method: HC / TTH- / TTH+: *p* < 0.001).**
Group differentiation based on T2 values	6	With average T2 as the predictor, the AUC for differentiating between the HC and TTH- groups was 0.68 (OR: 2.03, 95%-CI: 1.01–4.11, *p* = 0.04), and it was 0.82 for the differentiation between the HC and TTH + groups (OR: 3.80, 95%-CI: 1.36–10.61, *p* = 0.01). Furthermore, the AUC for differentiating between the TTH- and TTH + groups was 0.69 (OR: 1.61, 95%-CI: 0.73–3.53, *p* = 0.24).	With average T2 as the predictor, the AUC for differentiating between the HC and TTH- groups was 0.68 (OR: 2.03, 95%-CI: 1.01–4.11, p = 0.04), and it was 0.82 for the differentiation between the HC and TTH + groups (OR: 3.80, 95%-CI: 1.36–10.61, p = 0.01). Furthermore, the AUC for differentiating between the TTH- and TTH + groups was 0.69 (OR: 1.61, 95%-CI: 0.73–3.53, p = 0.24).

**Table 2:** Participant characteristics

	HC (*n* = 22)	vs. TTH- (*n* = 16)	*p*-value	vs. TTH+ (*n* = 12)	*p*-value
BMI (kg/m^2^) mean ± SD, range	22.**0** ± 2.3 19.1–28.7	22.**1** ± **2.4** 18.8–28.1	**0.843**	21.7 ± 1.7 18.9–24.7	0.689
No. of mTrPs mean ± SD, range	4.0 ± 4.0 0–**10**	3.8 ± 2.8 0–10	0.898	3.8 ± 2.**0** **1**–**8**	0.906
Headache (days/month) Mean ± SD, range	**2.0** ± 1.5 0–5 **(***n*** = 21)**	10.1 ± 7.6 3–30	< *0.001*	10.3 ± 6.**8** 4–25	*0.001*
Neck pain no. subjects	0 (0%)	10 (63%)	< *0.001*	**10** (83%)	< *0.001*

